# Contributions of decision science to ethical priority-setting in climate and health research

**DOI:** 10.2471/BLT.25.294063

**Published:** 2026-02-03

**Authors:** Neil Singh Bedi, S Skye Yoden, Ankur Pandya

**Affiliations:** a Center for Health Decision Science, Harvard T.H. Chan School of Public Health, 718 Huntington Ave, Boston, MA 02115, United States of America.

Climate change poses a major threat to human health.^1^ Despite the urgency to act, decision-making on climate in relation to health is inherently complex, characterized by incomplete, low-quality or absent data, economic resource constraints and ethical trade-offs. The nature and scale of climate impacts remain uncertain, such as who will be affected when, and which interventions will reduce harm.^2^ These challenges demand a systematic, transparent and value-informed approach to decision-making,^1^ such as that provided by decision science.

Decision science unites various fields of study and quantitative methods such as decision and risk analysis, benefit–cost and cost–effectiveness analysis, and simulation modelling to support rational, ethical decision-making in complex settings. At its core, we suggest that decision science involves a sequence of structured steps: (i) define the objectives and constraints; (ii) identify the available strategies; (iii) project outcomes under each strategy using the best available evidence; and (iv) make a decision.

Decision-makers, including those whose decisions affect climate change and its health consequences, use an implicit decision-making process. Adopting a decision science framing makes this process transparent, even when the underlying intentions of decision-makers are not made explicit. Here, we argue that a decision science framework coupled with a fair process can lead to legitimate climate-related policy decisions that quantitatively balance multiple objectives. We illustrate this argument through key decision science concepts of discounting future costs and benefits, and distributional cost–effectiveness analysis. The structured application of these two tools, which are already well established in health economics, bridges theoretical ethics and practical decision-making in climate and health.

## Decision science and fair process 

The objectives used in a decision analysis focus on consequences. While the selection of outcomes and weights assigned to them involve normative judgements, a decision analytic approach will consistently recommend the strategy that best meets its defined objective. A fair process, on the other hand, prioritizes procedural justice. Policy decisions are considered legitimate when the process meets certain fairness criteria. In public health, the prevailing accountability for reasonableness framework requires transparency, relevance (of evidence and stakeholder input), revisability (through an appeals process, for example) and accountability (decision-makers can be held accountable by voters, governments or peer institutions).^3^ Decision science and fair process frameworks are not mutually exclusive. In fact, they work best when used together.

When used as a prospective tool, decision science provides a systematic and transparent framework for navigating uncertainties, weighing relevant outcomes and directing the allocation of constrained resources. When used as a retrospective tool, decision science allows scrutinizing the climate-related decisions policy-makers make. Decision science can reveal the implicit value systems that shaped those decisions; with that transparency, decisions are opened to normative critique and accountability. This dual role enables decision science to function both as a compass and a mirror: guiding reasoned, transparent action while also holding systems and leaders accountable for their process and choices.

## Discounting future outcomes 

Standard practice in economic evaluations, whether in climate or health, is to apply discount rates consistently across health outcomes and costs, using economic market rates that are designed to function over 20- or 30-year cycles. However, many climate interventions generate health benefits over much longer periods, and these benefits can be lost in conventional analyses using standard discount rate values.^4^ Climate modelling studies have consistently found the discount rate to be an influential parameter, and routinely perform sensitivity analyses around this model input.^5^ We recommend that decision-makers (and decision-analysts) explicitly state the ethical positions and economic implications underlying their choice of discount rate values. For example, some national health technology assessment guidelines apply a higher annual discount rate to costs than to health outcomes (such as 4% for costs and 1.5% for health in the Kingdom of the Netherlands).^6^ This differential discount rate implies that future economic effects of policy choices are valued based on market valuations, but that future health outcomes are assessed differently and are not tied to these market returns. Another implication of using a lower discount rate for health than for cost outcomes is that, over time, health outcomes are effectively valued more highly relative to money.^7^ Whether or not differential discount rates should be used for climate-related decisions is beyond the scope of this article. Instead, we argue here that decision-makers should be transparent and accountable for the underlying rationale and implications for using specific discount rates, not just the numerical values themselves.

## Distributional analysis

Climate and health harms are felt unequally across populations worldwide.^8^ Heat waves, wildfire smoke pollution, floods and storms disproportionately affect specific communities, even within the same city or neighbourhood. This heterogeneity reflects random chance and histories of systemic oppression, colonization and structural inequity. Conventional cost–effectiveness analysis assumes all health gains and costs are valued equally across subpopulations, even if cost-effective resource allocations exacerbate existing health or economic disparities. Using country or subgroup-specific monetary valuations of health outcomes based on willingness-to-pay values, which will be greater for higher-income settings, can further amplify these disparities. Distributional cost–effectiveness analysis is a relatively new method being adopted within decision science that quantitatively captures the impacts of policy choices on distributional equity alongside the population-wide cost and health outcomes.^9^ The degree of inequality aversion (meaning the weight placed on distributional equity) can be based on population surveys or other deliberative exercises. A review of studies that assessed public attitudes towards health inequality found broad support for inequality aversion, that is, most respondents were willing to give up some total health for a more equal distribution of health.^10^ If these preferences are consistently popular across settings, democratic processes should hold decision-makers accountable for their choices on whether to account for inequality aversion and to what degree when making climate-related policy decisions. Just as for discounting, the underlying rationale for whether to choose an egalitarian approach such as distributional cost–effectiveness analysis, and if this choice is made, the inequality aversion parameter values, should be transparent.

## An intersection of discounting and equity 

While previous climate models have considered the impact of analytic choices such as the discount rate and distributional concerns, researchers and experts have focused less on how these elements can interact.^11^ Specifically, the combination of analytic choices around discounting and inequality aversion reveals the implicit values embedded in a decision (Table 1). For example, a decision analysis that uses high discount rates and high degrees of inequality aversion could reflect a rationale that improving the welfare of currently disadvantaged populations would improve the likelihood that future generations can adapt to and potentially mitigate climate-related health challenges. If these analytic choices and their rationales are made public, a fair process would hold decision-makers accountable for their positions, creating an incentive structure for adopting positions that reflect their constituents’ values.

**Table 1:** Normative values underlying degrees of discounting and inequality aversion

Inequality aversion level	Low discount rate	High discount rate
Minimal inequality aversion	Prioritizes an egalitarian distribution of welfare across generations (present and future), but maximizes total welfare within a generation	Prioritizes maximizing overall welfare in the present, with little to no consideration of the distribution of welfare across the population
High inequality aversion	Prioritizes egalitarian distribution of welfare both across and within generations	Prioritizes egalitarian distribution of welfare within the present, with less consideration for future generations

Discounting and distributional cost–effectiveness analysis are just two elements within a decision science framework that we highlight here because of their relevance to climate and health decision-making. Decision-makers can and should be equally transparent about their rationale and implications for other aspects of their approach, such as willingness-to-pay for health values, estimates for social cost of carbon, and the ways in which uncertainty and risk aversion are modelled, particularly for low-probability but potentially catastrophic events.

## Challenges and opportunities 

Adopting either a decision science framework or fair process, or both, to inform climate-related policy decisions requires the participation of good-faith actors and well-functioning political environments. Both requirements seem aspirational considering recent global events, where good-faith discussions are increasingly constrained by mutually exclusive political strategies, and many governments have shown signs of movement away from democratic principles towards nationalistic and authoritarian regimes. Data limitations further constrain capacity; many countries lack consistent, high-resolution and disaggregated data sets linking health outcomes to climate variables, and data on the efficacy of certain interventions are limited. Corporate interests also loom over many climate-related decisions, creating the incentives for performing and promoting biased decision analyses through misinformation or black-box modelling tactics. Decision science methods or fair processes cannot by themselves remove these broader obstacles from climate and health policy-making.

We believe, however, that using decision science as a retrospective tool will help individuals and communities link a leader’s decisions to their underlying values and beliefs. Furthermore, many individuals and communities seek meaningful solutions and better systems to combat climate change and its downstream impacts on human health and livelihoods.^12^ The field of decision science can support local actors with decision analysis and fair process frameworks, and help those actors understand what values and beliefs they are embedding into their prospective models. Our approach will not lead to perfect consensus on the value judgments that ultimately inform decisions on the path forward. Rather, combining decision science methods with fair process structures facilitates these discussions in a legitimate and rigorous way. Decision science offers the tools to map individual, community, national and global value systems to decisions involving hard trade-offs, helping to inform policy choices that promote health and well-being for all.
